# Case Report: A management strategy and clinical analysis of primary squamous cell carcinoma of the colon

**DOI:** 10.3389/fonc.2023.1265421

**Published:** 2023-10-11

**Authors:** Xiang Wu, Shenyong Su, Yaning Wei, Dan Hong, Zhiyu Wang

**Affiliations:** Department of Medical Oncology, Affiliated Hospital of Hebei University, Baoding, China

**Keywords:** colorectal cancer, squamous cell carcinoma, adjuvant chemotherapy, immune checkpoint inhibitors, microsatellite instability, case report

## Abstract

Primary colorectal squamous cell carcinoma (CSCC) is a rare pathological subtype. Currently, clinical data with regards to its prognosis and treatment is limited, and there is no optimal treatment method. The case presented involves a proficient mismatch repair (pMMR) and microsatellite-stable (MSS) Colorectal cancer (CRC) patient with squamous cell carcinoma (SCC) located transversely in the colon. Based on the imaging assessment, the tumor infiltration depth is classified as T4. After receiving 4 cycles of neoadjuvant treatment with oxaliplatin and capecitabine (XELOX), the patients were evaluated for partial response (PR) in 2 cycles and stable disease (SD) in 4 cycles. The patient underwent a right hemicolectomy and received postoperative paclitaxel/cisplatin (TC) adjuvant chemotherapy. After 23 months, a systemic examination revealed abdominal metastasis. A needle biopsy was conducted on the detected abdominal metastases, with the resulting pathology indicating the presence of metastatic SCC. The individual exhibited expression of programmed cell death ligand 1 (PD-L1) and a mutation in the TP53 gene. Considering the patient’s disease recurrence based on medical history, a treatment plan was formulated. This involved Sintilimab plus Cetuximab and the combination of leucovorin, fluorouracil, and irinotecan (FOLFIRI) regimen. The patient received four cycles of treatment with an efficacy evaluation of SD- and seven cycles of treatment with an efficacy evaluation of SD+, which resulted in a progression-free survival (PFS) duration of 7 months. This case study presents the conventional XELOX chemotherapy protocol, which has shown limited effectiveness, and highlights the favorable results achieved by implementing the TC adjuvant chemotherapy regimen in individuals diagnosed with primary colonic SCC. Furthermore, combining immune checkpoint blockade (ICB) with other therapies for patients with advanced disease is anticipated to provide an extended duration of survival.

## 1 Introduction

Primary CSCC is an infrequent form of tumor, representing a mere 0.01-0.025% of the total cases of colorectal cancer (1). The mean age of the patients was 63.5 ± 15.3, with no significant difference in incidence between men and women (2). The most common site of occurrence was the rectum, followed by the right colon (3). The majority of cases were found to be complicated by lymph node and liver metastases (4). The patient’s clinical presentation resembled that of colorectal adenocarcinoma, with nearly a half of patients displaying symptoms of gastrointestinal bleeding or abdominal distress (3, 5). The initial diagnosis is mostly advanced, with a poor prognosis (6). Usually, patients with metastasis in distant organs have a median survival rate of about 8 months (7). The five-year relative survival rate is notably inferior compared to that of colorectal adenocarcinoma (2).

Patients with primary rectal SCC are mainly treated with a combination of surgical intervention and radio chemotherapy (4, 7). Significantly, primary rectal SCC at stage II manifests a high sensitivity to chemoradiotherapy, and the administration of neoadjuvant chemoradiotherapy in patients prior to surgery has demonstrated a positive correlation with improved survival rates (8). Patients diagnosed with stage III or IV rectal SCC are typically treated through a combination of radiotherapy and chemotherapy; adding surgical interventions concurrently does not improve the overall survival (OS) of these patients (1). However, for patients experiencing recurrence or an ineffective response to radiotherapy and chemotherapy, the option of surgical intervention is available (9).

Divergent from rectal SCC, the most important treatment for patients with primary colonic SCC is surgery, and the efficacy of chemotherapy or radiotherapy is still unclear (10). Surgical intervention is the primary therapeutic modality employed for patients diagnosed with colonic SCC in Stage II (11). In cases of stage III colonic SCC, the treatment regimen involves a combination of surgical intervention and chemotherapy. In general, patients diagnosed with this condition commonly receive fluorouracil with or without cisplatin adjuvant chemotherapy (12, 13). Palliative treatment is the main approach for patients with metastatic primary colonic SCC. Hence, it is imperative to engage in further discourse regarding the management of patients diagnosed with colonic SCC.

## 2 Case presentation

**Figure 1:**
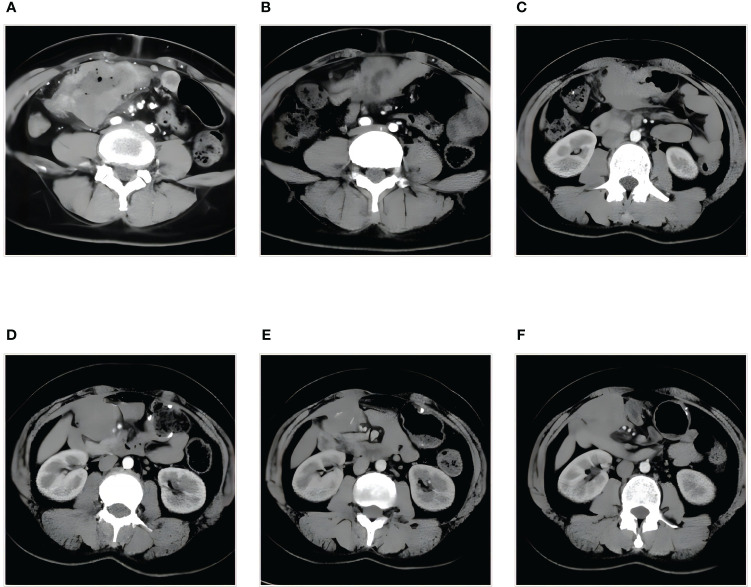
Treatment assessment by abdominal enhanced CT **(A-F)**. **(A)** Diagnosis, the tumor infiltration depth is classified as T4. **(B)** PR, after two cycles of XELOX therapy. **(C)** SD, after four cycles of XELOX therapy. **(D)** PD, abdominal metastasis. **(E)** SD-, after four cycles of combination therapy. **(F)** SD+, after seven cycles of combination therapy. PR, partial response; PD, progressive disease; SD, stable disease.

**Figure 2:**
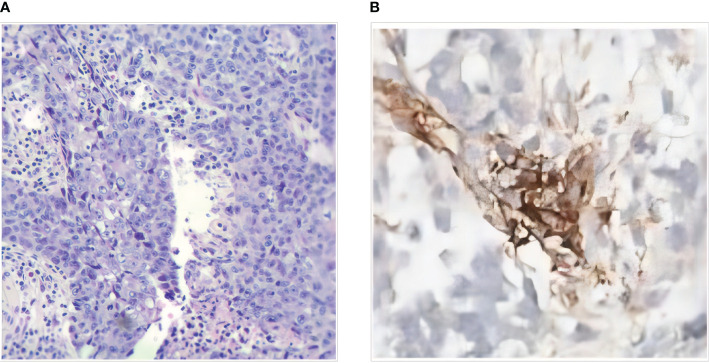
**(A)** Histopathology of SCC of transverse colon (HE×100). **(B)** Immunohistochemistry (IHC) of the Abdominal nodule biopsies.

The 41-year-old female patient presented to the clinic with abdominal pain and was diagnosed with colon cancer on January 16, 2020 (**Figure 1A**). Colonoscopy, the pathological results of the biopsy, and immunohistochemistry (IHC) indicated poorly differentiated SCC located in the transverse colon. The patient received a comprehensive examination that ruled out the presence of distant metastases and primary tumors. The imaging assessment results indicate that the tumor infiltration depth was classified as T4, suggesting local progression of the disease. As a result, neoadjuvant treatment was administered. The XELOX regimen was utilized for therapy, which was then followed by 2 cycles of treatment with a PR efficacy evaluation (**Figure 1B**). Subsequently, 4 cycles of treatment were given with an efficacy evaluation of SD (**Figure 1C**). The MDT (Multidisciplinary Team) that deliberated on the case after the neoadjuvant treatment concluded that surgical resection of the neoplasia had become feasible. The patient underwent a right hemicolectomy procedure on May 22, 2020. The postoperative pathological examination yielded a poorly differentiated SCC located in the colon (**Figure 2A**). The tumor’s largest diameter measured 6.5cm. The tumor infiltrated the muscular layer and reached the subserosal fibrous adipose tissue. The malignancy was visible at the circumferential cutting edge, and no clear vascular tumor thrombus or nerve infiltration was found, which was in line with the chemotherapy response (AJCC/TRG grade 3). No metastatic cancer was found in lymph nodes (0/43). The IHC indicated BRAFV600E (-), PMS 2 (+), MLH 1 (+), MSH 2 (+), MSH 6 (+), CD56 (-), Syn (focal weak +), CgA (+), CK20 (partial +), CDX 2 (+), P40 (+), P63 (+), and Ki-67 (70%). Between June 25th, 2020, and November 30th, 2020, the patient commenced treatment with the TC adjuvant chemotherapy protocol. The patients were then regularly monitored, and the medical condition remained stable.

**Figure 3:**
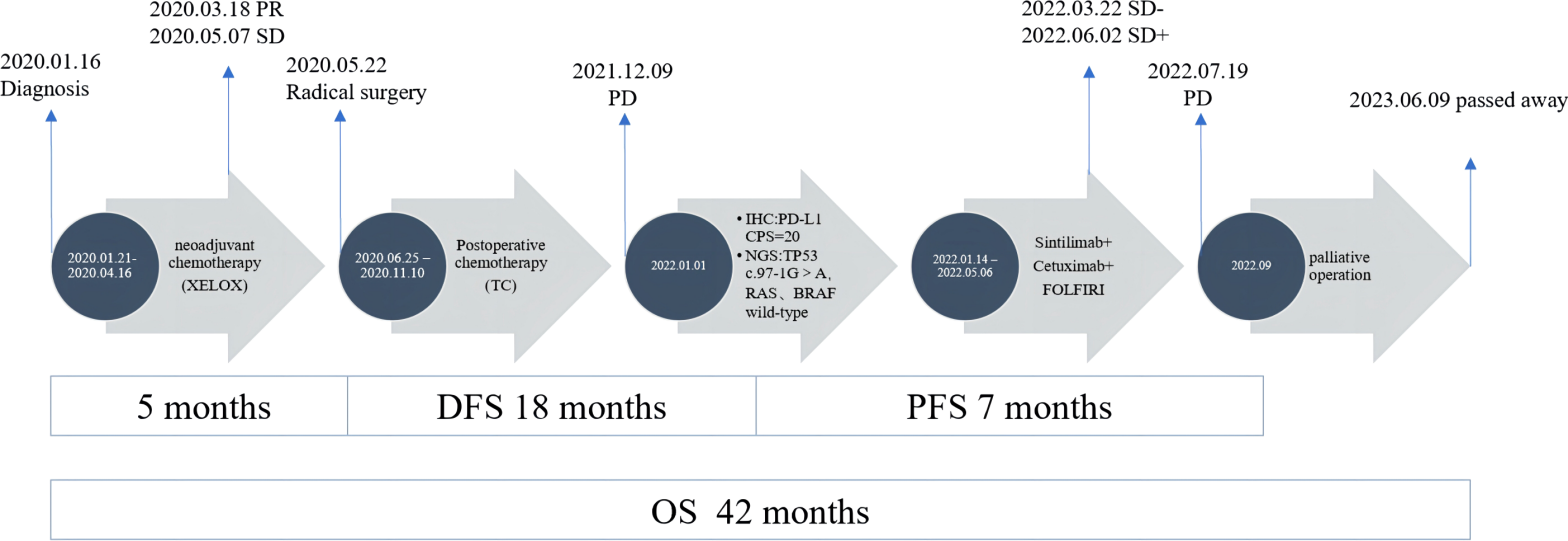
Treatment summary of the patient from diagnosis to last follow-up. PR, partial response; PD, progressive disease; SD, stable disease.

On December 9, 2021, a mass located in the right upper quadrant of the anterior abdominal wall was observed during the reexamination of an abdominal enhanced CT (**Figure 1D**). On December 23, 2021, the abdominal mass was subjected to a CT-guided percutaneous needle biopsy. The pathology report suggested the possibility of metastatic SCC. Genetic testing revealed a mutation in the TP53 gene, but RAS and BRAF were wild-type, and IHC indicated that the patient had PD-L1: CPS (Combined Positive Score) = 20 (**Figure 2B**). On January 14, 2022, she was treated with Sintilimab plus Cetuximab and the FOLFIRI regimen. The patient received four cycles of treatment with an efficacy evaluation of SD- (**Figure 1E**) and seven cycles of treatment with an efficacy evaluation of SD+ (**Figure 1F**). The patient’s review on July 19, 2022, revealed PD in the condition. The patient’s PFS was approximately 7 months. Considering that the patient is progressing with oligolesions, there is presently a lack of a standardized second-line treatment plan with limited effectiveness. Considering that the patient was progressing by developing oligolesions, there did not, at the time, exist a standardized second-line treatment plan with a certain degree of effectiveness. In September of the same year, the patient underwent a surgical procedure to remove the abdominal mass. Postoperative pathological consideration revealed metastatic SCC. Subsequently, the patient did not receive any further anti-tumor treatment. Regrettably, the patient passed away on June 9, 2023. (**Figure 3**).

## 3 Discussion

As a rare pathological type, primary CSCC currently has no established standard treatment available. Most of the studies were focused on rectal SCC, with limited research available on colonic SCC. There was no comprehensive and systematic evidence-based study on its treatment regimen and survival prognosis. Most of the information comes from individual case reports. The present article presents the case of a patient who initially received a diagnosis of locally advanced SCC of the transverse colon. However, the surgical evaluation did not result in an R0 resection. Consequently, the patient underwent four cycles of neoadjuvant chemotherapy, followed by a right hemicolectomy procedure. Postoperative pathology suggested an AJCC/TRG grading of 3. The patient was administered adjuvant chemotherapy with TC with a PFS of 18 months. According to reports, the TC systemic chemotherapy regimen has shown superior treatment outcomes when compared to the 5-fluorouracil and cisplatin (FP) regimen for the management of esophageal squamous cell carcinoma (ESCC) in patients who receive adjuvant chemotherapy after surgery (14). In the postoperative treatment of head and neck squamous cell carcinoma (HNSCC), TC combined with radiotherapy improves the disease control rate (DCR) (15). Meanwhile, the utilization of TC in postoperative settings has been found to extend the period of disease-free survival (DFS) among patients diagnosed with cervical SCC (16). The TC regimen for individuals diagnosed with SCC has some clinical benefits. In the case of colonic SCC, it may be worth comparing the effectiveness of TC and FP.

Hence, given its unique pathological characteristics, CSCC appears to require a distinct approach to treatment when compared to colon adenocarcinoma. Currently, the accepted adjuvant therapy protocol for colon adenocarcinoma consists of the XELOX, mFOLFOX6 (oxaliplatin, fluorouracil, and leucovorin), etc. As per the standard treatment protocol, patients diagnosed with colon adenocarcinoma have shown a 3-year PFS rate of 76% (17). The primary approach for treating patients with advanced colon adenocarcinoma continues to be chemotherapy, plus Bevacizumab or Cetuximab is a feasible treatment option (18, 19). Immunotherapy has shown limited efficacy in treating gastrointestinal tumors, particularly in patients with colon cancer. However, it has been observed that Pembrolizumab is effective as a first-line treatment for metastatic colon cancer patients who have MSI-H or dMMR (20, 21). In recent times, it has been put forth the notion that the fusion of ICB and other treatment methods could emerge as a novel therapeutic choice for pMMR or MSS CRC (22). Research has demonstrated that Avelumab and Cetuximab possess complementary modes of operation that can effectively collaborate to counter the negative feedback of immunosuppression through synergistic action (23). The joint utilization of Pembrolizumab and Cetuximab results in a synergistic antitumor outcome by promoting a more advantageous anti-tumor microenvironment via the amplification of intracellular cytotoxic T lymphocytes and NK cells (24). The phase II CAVE clinical trial findings indicate that the combination of Cetuximab and Avelumab effectively targets patients with MSS metastatic CRC, exhibiting significant rechallenge therapy (25). Incorporating Avelumab into the treatment regimen consisting of Cetuximab and chemotherapy resulted in a noteworthy increase in the objective response rate (ORR) to 83% among patients with MSS CRC (26).

The current case report showcases a patient diagnosed with colonic SCC and exhibiting PD-L1 expression. After experiencing disease relapse, the patient underwent treatment with a regimen consisting of Sintelimab, Cetuximab, and chemotherapy. As a result, the patient achieved a PFS of 7 months. In the palliative treatment of colonic SCC, there are individual case reports indicating that immunotherapy can be used for these patients with PD-L1 expression. A case of a patient diagnosed with pMMR/MSS primary rectosigmoid-junction SCC and presented with high PD-L1 (CPS = 60) expression and tumor mutational burden (TMB-High, 18.99 mutations/mb), received Sintilimab combined with chemotherapy, and obtained a disease-stabilizing period of one year (27). A case of a patient suffering from pMMR/MSS primary colonic SCC with high PD-L1 (CPS = 20) expression underwent treatment involving Sintilimab combined with mFOLFOX6 and achieved a PFS of 8.5 months (28). According to previous data, the median OS for patients with advanced colonic SCC who only received chemotherapy was approximately 8 months (7). It is evident that immunotherapy treatment for colon SCC offers a survival advantage, possibly linked to the expression of PD-L1.

Based on preclinical experiments, it has been demonstrated that SCC with PD-L1 expression can be effectively suppressed through the use of ICB (29). The expression of PD-L1 on tumors reflects an immunocompetent microenvironment and is considered a major factor in anti-PD-1 therapy (30). And research has shown that the expression of PD-L1 on tumor cells is directly proportional to the response to ICB (31, 32). According to previous reports, the expression of PD-L1 is found in diverse solid tumors, encompassing lung, esophageal, and head and neck squamous cell carcinomas (33). However, there is limited available data on the expression of PD-L1 in colonic SCC.

In addition, immune response as a potential target for the treatment of SCC is associated with distinct gene expression profiles (34). The different mRNA expression patterns suggest that each SCC possesses unique immune signatures (35). Song et al. analyzed the proteome of SCC cancers from 17 organs and identified six distinct immune subtypes of pan SCC, each exhibiting unique tumor microenvironment (TME) characteristics and varying prognostic outcomes. However, it is worth noting that these samples contain common and rare sites of SCC, but do not involve the colon (36). Therefore, there is still no good description of the molecular mechanism of CSCC.

According to this report, it is suggested that the use of TC as an adjuvant chemotherapy regimen may exhibit favorable anti-tumor effects when treating colonic SCC. Additionally, the report recommends exploring the potential of ICB when used in conjunction with other therapies for treating patients with progressive colonic SCC. And the expression level of PD-L1 could be used as a biomarker for the application of ICB therapy in patients.

## Data availability statement

The raw data supporting the conclusions of this article will be made available by the authors, without undue reservation.

## Ethics statement

The studies involving humans were approved by Affiliated Hospital of Hebei University. The studies were conducted in accordance with the local legislation and institutional requirements. The participants provided their written informed consent to participate in this study. Written informed consent was obtained from the individual(s) for the publication of any potentially identifiable images or data included in this article.

## Author contributions

XW: Writing – original draft. SS: Writing – review & editing. YW: Writing – review & editing. DH: Writing – review & editing. ZW: Writing – review & editing.
